# Wireless Sensors System for Stress Detection by Means of ECG and EDA Acquisition

**DOI:** 10.3390/s20072026

**Published:** 2020-04-04

**Authors:** Antonio Affanni

**Affiliations:** Polytechnic Department of Engineering and Architecture (DPIA), University of Udine, Via delle Scienze 206, 33100 Udine, Italy; antonio.affanni@uniud.it

**Keywords:** electrodermal activity, stress measurement, ECG sensor, driving simulators

## Abstract

This paper describes the design of a two channels electrodermal activity (EDA) sensor and two channels electrocardiogram (ECG) sensor. The EDA sensors acquire data on the hands and transmit them to the ECG sensor with wireless WiFi communication for increased wearability. The sensors system acquires two EDA channels to improve the removal of motion artifacts that take place if EDA is measured on individuals who need to move their hands in their activities. The ECG channels are acquired on the chest and the ECG sensor is responsible for aligning the two ECG traces with the received packets from EDA sensors; the ECG sensor sends via WiFi the aligned packets to a laptop for real time plot and data storage. The metrological characterization showed high-level performances in terms of linearity and jitter; the delays introduced by the wireless transmission from EDA to ECG sensor have been proved to be negligible for the present application.

## 1. Introduction

During recent years, the interest of scientific literature in the measurement of the stress in individuals has increased exponentially. Just in the current year, hundreds of works have been published with the aim of investigating the stress induced by several conditions of every-day life. Some works aim at the measurement of stress in the workplace [1], some others investigate stress while people are playing videogames [2], but the majority of studies investigate the mental stress while people are driving [3,4,5,6,7,8,9,10].

In recent literature [6,11], there have been several works which study driver attention by means of computer vision and eye-tracking systems, but these papers are mainly focused on mental workload and attention of the driver; it has been demonstrated in this field of research that pupil diameter measurement can be correlated to cognitive load of the driver in recognizing obstacles and pedestrians [12]. However, the mental stress of the driver seems to be poorly correlated to pupil diameter or fixation duration [13] and, moreover, the eye-tracking systems need complex and accurate calibration on each subject under test. For these reasons, currently the majority of scientific papers focusing on driver mental stress detection rely on the measurement of bio-signals.

In order to detect the mental stress level of a person, several bio-signals are measured; in Reference [14] photoplethysmogram (PPG) is acquired, in Reference [3] the authors measure the electroencephalogram (EEG), but the electrocardiogram (ECG), with the consequent heart rate variability (HRV), combined with electrodermal activity (EDA) are the most used methodologies [1,4,15,16,17,18,19,20,21,22]. The combination of HRV and EDA methodologies is also widely used to train machine learning algorithms able to detect the level of stress; in the literature, the detection accuracy of stress, obtained from ECG and EDA signals, is in the order of 80% [23,24,25,26]. Moreover, in the recent past there was an increase in development of new sensors (either for academic or commercial purposes) able to measure the EDA with wearable devices having low invasiveness [27,28,29].

However, the main problem of using different commercial devices (having different protocols and data storage) is the capability of aligning and fusing data with an acceptable accuracy in an automatic way, since the timebase of each sensor is different. In the recent past, we developed a device measuring three channels ECG on the chest and one channel EDA on the hand [16], but the most critical point was to connect wires from the chest to the hand. So, we moved to a device which acquired two channels EDA and one channel ECG that had electrodes only on the hands [20]; however the signal to noise ratio was lower (with respect to ECG acquired on the chest) and during driving sessions (especially in simulators on Formula 1 tracks) some artifacts were recorded. So, in this paper we reintroduce the chest derivations for ECG measurement because we have the main focus on studying the mental stress of professional drivers on dynamic simulators; the importance of their psycho-physiological state during simulator sessions is of paramount importance for car factories to develop new cars without producing physical car prototypes. The dynamic model of a new car (mass distribution, springs, dampers...) is in fact simulated and, on the basis of drivers impressions, modified without creating an expensive physical prototype of the car. In this scenario, we want to add an objective indicator of mental stress to the written questionnaires currently filled by drivers after each driving session.

Even if our main research activity focuses on mental stress detection of drivers at the simulator, the sensor developed in the present paper can be applied also to other stressing conditions, for example in the literature we find application of EDA and ECG to human-robot interaction [30], but also to stress detection in the workplace [31,32]. However, the use of chest electrodes may be a little bit uncomfortable in everyday life, especially for non-professional drivers who use the car just for daily motion; in a simulator environment instead, professional drivers do not perceive the data acquisition with vest and gloves as an uncomfortable situation.

In this work we describe the design of a low cost sensor for mental stress detection, measuring two EDA channels on the hands and two ECG channels on the chest, using a completely wireless transmission from the hands to the chest and from the chest to a laptop; in this way, data packets are always aligned since they are sent from a unique sensor. In Reference [20] we also pointed out the need of two channels for EDA measurement in driving scenarios since the motion of the hands (where EDA electrodes are placed) on the steering wheel gives rise to a significant motion artifact, which makes a single channel EDA sensor quite unreliable because it is very difficult to separate the motion artifact from sympathetic activity [18]. Moreover, most of the studies in the literature make use of exosomatic EDA methodology (namely skin conductance response (SCR) or galvanic skin response GSR), where a current is injected into the body and a conductance information is extracted; in our studies, we concentrate instead on endosomatic EDA (namely skin potential response (SPR)) because it has been proved in the literature [33,34] that SPR is faster than SCR; so, when evaluating the instantaneous stress of a driver when immersed in a scenario having sudden stimuli, SPR provides a more punctual information on the sympathetic activity. Unfortunately, at the moment on the market there are no commercial solutions for the measurement of endosomatic EDA by means of wearable devices.

SPR directly acquires the nervous pulse which activates the sweat glands, thus the conditioning circuitry consists of a differential amplifier (like in the case of ECG, but with different gain and bandwidth); this methodology implies more complex signals, but the sensor readout is faster and it is less prone to electrode impedance changes due to sweating of the hands or to temperature variations. SPR signal manifests as a differential voltage when we apply one electrode in a zone with a high concentration of sweat glands (e.g., the palm of the hand) and another electrode where sweat glands are fewer (e.g., the back of the hand). In the recent literature (e.g., in Reference [21]), we find several papers where EDA and ECG are simultaneously acquired, also with commercial solutions, but ECG data are not automatically aligned with EDA because data are coming from different commercial sensors having different timebase; moreover, EDA is acquired by means of SCR, which provides a slower reaction to stimuli.

The measurement of HRV has been described in hundreds of scientific papers published in the last decade. The heart rate variability is modulated by the sympathetic nervous system and by the parasympathetic (vagal) nervous system; these two systems control HRV with different speeds of intervention: sympathetic system varies HRV slowly, while vagal varies HRV quickly. The processing methodology of HRV is still an open issue in the literature; some features are extracted in frequency domain (Low Frequency power LF, High Frequency power HF, and their ratio LF/HF), some others in time domain (Standard Deviation Normal to Normal SDNN, Root Mean Square of Successive Differences RMSSD), and newer features are moving towards symbolic approaches, especially for short time acquisitions [35,36,37]. Since SPR gives valuable information about short-term sympathetic activity, the simultaneous acquisition of HRV and EDA can help to improve the methodologies to extract HRV features related to sympathetic activity, especially in short term evaluation.

In a recent paper [26], we proved that the combination of SPR and ECG signals can provide several features for machine learning algorithms, like Support Vector Machine (SVM) and Artificial Neural Networks (ANN), which can discriminate mental stress on drivers in a simulator. In that paper, 16 healthy individuals took part in the test (performed on a professional dynamic driving simulator) and had to drive in a simulated 28 km long highway, with four obstacles, requiring some effort to cross, positioned at different distances along the road; all the intervals belonging to a road section with obstacles were supposed to be “1”, with stress, and all the intervals belonging to a section without obstacles were supposed to be “0”, without stress.. From the SPR signal we extracted five features—variance, energy, mean absolute value, mean absolute derivative and max absolute derivative. From the ECG signal we extracted eight features—the mean value of normal-to-normal RR (or NN) intervals, the standard deviation of RR intervals (SDNN), the standard deviation of successive RR interval differences (SDSD), the root mean square of successive RR interval differences (RMSSD), the number of successive RR intervals differing more than 50 ms (NN50) and the corresponding value in percentage (PNN50), the mean value of the Heart Rate (HR) and the HR mean derivative value. The experimental data demonstrated that both SVM and ANN recognized the mental condition with accuracy, specificity and sensitivity higher than 75%. The sensor that we used was that described in Reference [16], but, as stated before, there was the need to connect wires from the hands to the chest, so the wearability needed to be improved.

The aim of the present paper is the design and metrological characterization of a wearable, fully wireless system which can acquire ECG and SPR signals with the same accuracy of a wired sensor. In particular, the key point is to assure a reliable transmission with no delays or data losses because the two SPR signals must be aligned with each other and both must be aligned with ECG.

The paper is organized as follows—Section 2 describes the architecture of the sensor, with particular attention to the description of the alignment of data packets sent from SPR sensor to ECG sensor; Section 3 describes the metrological characterization of the sensors using three different approaches to evaluate the accuracy of the developed device—a first approach characterizes the linearity and bandwidth, the second approach evaluates jitter using a synthesized ECG trace and a third approach compares ECG acquisitions simultaneously with a commercial reference sensor to quantify the accuracy in the extraction of main features used in HRV. At the end of Section 3, we show an example of an application of the present sensor to detect the stress of subjects driving on a simulator available in our laboratory at the University of Udine. Finally, after Discussion, the Conclusions are drawn.

## 2. Material and Methods

**Figure 1:**
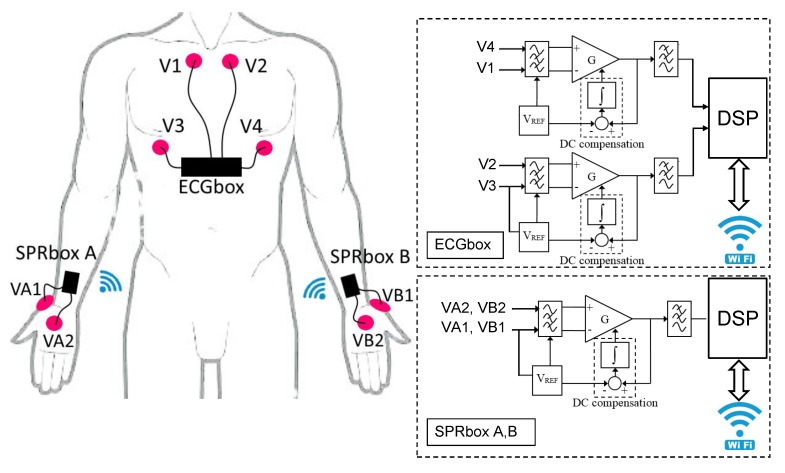
(**Left**) scheme of sensors and electrodes positioning—the skin potential response (SPR) boxes A and B acquire electrodermal activity (EDA) signals on the hands and transmit the data to the electrocardiogram (ECG) box; the ECG box acquires signals on the chest and sends data to a laptop. (**Right**) block diagram of SPR boxes and ECG box.

The entire wearable sensors system is shown in Figure 1. The SPRbox A acquires the SPR on the right hand where two disposable adhesive Ag/AgCl electrodes are posed on the palm and on the back. The reference potential $V_{REF}$ is imposed on the VA1 electrode and the differential voltage VA2−VA1 is amplified and conditioned, acquired by an A/D converter on board a Digital Signal Processor (DSP) and sent to the ECGbox via wireless standard IEEE 802.11 (WiFi) using User Datagram Protocol (UDP). In the same way, on the left hand, the SPRbox B conditions acquires and sends the differential voltage VB2−VB1 to the ECGbox via WiFi UDP protocol. The ECGbox is responsible for conditioning and acquiring two ECG derivations, to receive and re-order the packets from SPRbox A and B, and to transmit the aligned data to a laptop via WiFi UDP protocol. In particular, the ECG traces are obtained conditioning and amplifying the differential voltages V4−V1 and V2−V3 on the chest, acquired with four disposable adhesive Ag/AgCl electrodes. Referring to Figure 1, it is possible to see that the block diagram of the ECGbox consists of two analog front ends, one DSP and a WiFi module; in a similar way, SPRbox A and SPRbox B consist of an analog front end, a DSP and a WiFi module.

The SPRbox A and B use single cell 3.7 V 850 mAh lithium polymer batteries which are rechargeable through a micro-USB plug. Since the current consumption of SPR boxes is 85 mA during transmission, the sensors can transmit data for 10 hours of continuous operation. The ECGbox is supplied with a single cell 3.7 V 2000 mAh lithium polymer battery and it is rechargeable through a micro-USB plug. Since the current consumption of the ECGbox is 180 mA during transmission, the sensor can transmit data for more than 11 hours of continuous operation.

**Figure 2:**
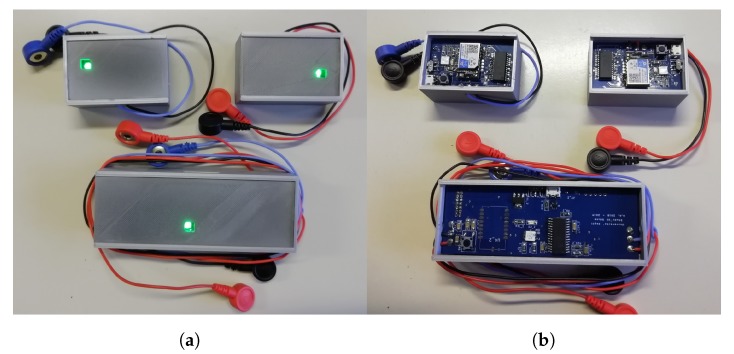
(**a**) Enclosures of SPR boxes and ECG box, and (**b**) Printed Circuit Boards of the developed sensors.

Figure 2a shows the boxes inside the enclosures, realized with 3D-printing technology; Figure 2b shows the Printed Circuit Boards of ECGbox and SPRboxes. The outer dimensions of the SPRbox are 55 × 37 ${\mathrm{mm}}^{2}$, while the ECGbox outer dimensions are 108 × 41 ${\mathrm{mm}}^{2}$; we observe that the outer dimensions are limited by the size of the batteries, so the boxes and the boards dimensions can be strongly shrunk if we decide to reduce the maximum duration of transmission. The SPR boxes are worn on wrists using velcro straps (like wearing a smart-watch) in order to keep the sensors firmly and unobtrusively adherent to the body, while the ECG box is worn with an elastic chest strap.

### 2.1. Analog Section

Referring to Figure 1, the analog front ends measure low-level differential voltages and, through a proper signal conditioning, provide a high level voltage which can be acquired by the A/D converter on board the DSP. The input differential voltages are filtered with passive, first order, high-pass filters (with cutoff frequency ${\left(2\pi \tau_{L}\right)}^{-1}=0.05$ Hz). Since the output impedance of the skin is in the order of 100 kΩ, the input impedance of the filters is 100 MΩ—three orders of magnitude higher—in order to reduce the load uncertainty to less than 0.1%. Cascaded to the input filter there is an instrumentation amplifier with gain $G_{SPR}$ and $G_{ECG}$ (for SPR and ECG channels, respectively) which amplifies the input differential signals and, at the output of the amplifier, there is a third order, Butterworth, anti-alias filter with time constants $\tau_{H,SPR}=10\;\mathrm{ms}$ and $\tau_{H,ECG}=1\;\mathrm{ms}$ for SPR and ECG signals, respectively; thus, the expected 3 dB cutoff frequencies of the third order filters are 8 Hz and 75 Hz for SPR and ECG, respectively. The DC compensation block integrates (with time constant designed $\tau_{I}=\tau_{L}$) the difference between the amplifier output and $V_{REF}$. Thus, the closed loop composed by the amplifier and the DC compensation block behaves as a high pass filter with cut-off frequency ${\left(2\pi \tau_{L}\right)}^{-1}$. Using this DC compensation block, we are able to remove all the DC non-idealities of the amplifier (offset voltage, bias current, offset current) [29]. The output voltage of the analog front end (for SPRbox and ECGbox, respectively), in the Fourier domain, is

(1) $$V_{SPRA,B}\left(j\omega \right)=2\pi V_{REF}\cdot \delta \left(\omega \right)+\frac{G_{SPR}\tau_{L,SPR}^{2}\cdot {\left(j\omega \right)}^{2}}{{\left(1+\tau_{L,SPR}\cdot j\omega \right)}^{2}{\left(1+\tau_{H,SPR}\cdot j\omega \right)}^{3}}\cdot \left(V_{A2,B2}\left(j\omega \right)-V_{A1,B1}\left(j\omega \right)\right),$$

(2) $$V_{ECG1,2}\left(j\omega \right)=2\pi V_{REF}\cdot \delta \left(\omega \right)+\frac{G_{ECG}\tau_{L,ECG}^{2}\cdot {\left(j\omega \right)}^{2}}{{\left(1+\tau_{L,ECG}\cdot j\omega \right)}^{2}{\left(1+\tau_{H,ECG}\cdot j\omega \right)}^{3}}\cdot \left(V_{4,2}\left(j\omega \right)-V_{1,3}\left(j\omega \right)\right).$$

We set $V_{REF}=1.65$ V at one half of the supply range that is 3.3 V. The gain of the amplifier of the SPRbox has been set $G_{SPR}\approx 160$, since the input range is ±10 mV and it is converted into 3.3 $V_{PP}$; the SPRbox has a band pass frequency response in the range [0.08, 8] Hz with slopes +40 dB/dec for the lower cut-off and –60 dB/dec for the higher cut-off. The gain of the amplifiers of the ECGbox has been set at $G_{ECG}\approx 370$, since the input range is ±5 mV and it is converted into 3.3 $V_{PP}$; the ECGbox has a band pass frequency response in the range [0.08, 75] Hz with slopes +40 dB/dec for the lower cut-off and −60 dB/dec for the higher cut-off.

### 2.2. DSP and A/D Conversion

The SPR and ECG signals, after the conditioning of analog front end, are acquired by the A/D input of a Digital Signal Processor (DSP), which processes the data (the processing is described in next Section) and sends them to the WiFi module via a Universal Asynchronous Receiver Transmitter (UART). The chosen DSP is a Microchip DSPIC 30F3013, it operates at 8 MIPS and has an on board 12 bit Analog-to-Digital Converter; the sampling frequency is set to 200 Hz, this value is high enough to record ECG signals with low jitter on the peak detection for HRV analysis, without sending too many data to a medium-low performance laptop. The sampling and conversion time of the A/D has been set to 50 μs, so that the sampling transients affect the converted data by far lower than 1 LSB. The baud rate of data transfer has been set to 115.2 kbps in order to allow data transmission without crowding the channel.

### 2.3. Data Transmission: SPRbox

**Figure 3:**
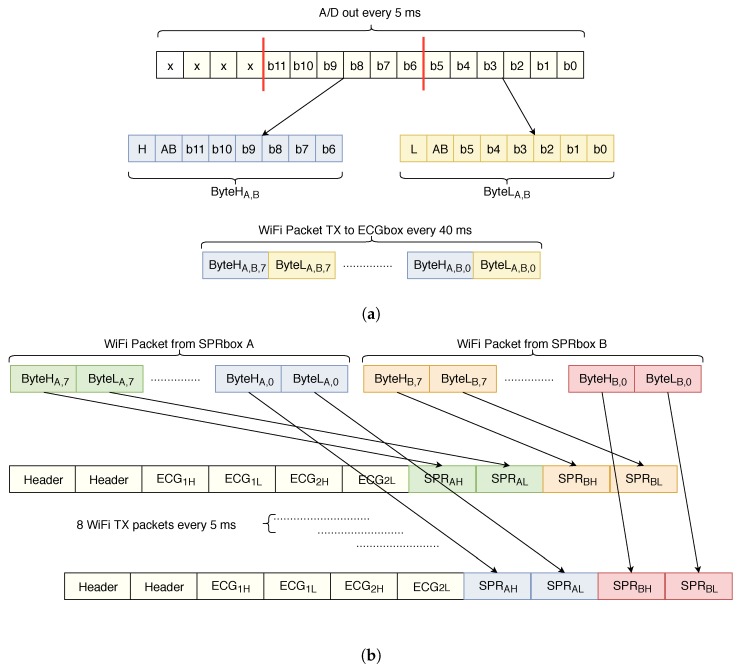
(**a**) Packets generation for transmission from SPR boxes to ECG box, and (**b**) packets alignment and reconstruction performed by the ECG box.

Figure 3a shows how the packets are built by the DSP on the SPRbox before transmission. The A/D module provides a 12 bit datum every 5 ms; each byte sent via UART to the ECGbox must be identified with a unique coding, since the ECGbox must recognize if the incoming datum is the higher or lower byte of the SPR box A or B. So, the DSP of SPRbox builds the lower (higher) byte of information using the six least (most) significant bits of the A/D, adding one bit for left/right box (AB bit in Figure 3a) and one bit for lower (higher) byte (L or H bit in Figure 3a).

The chosen WiFi module is the USR-IOT USR-C216; it is a low power module (60 mA consumption during transmission) and, for consumption reasons, it can send packets every 40 ms minimum. For this reason, the DSP builds a packet composed by eight data acquired every 5 ms and sends them to the ECGbox. The module is configured as station (STA) with static IP and operates as a client UDP; the gateway address is configured to be the one of ECGbox, in order to send SPR data to the ECGbox which re-aligns the data acquired by SPRbox A and B.

### 2.4. Data Transmission: ECGbox

Figure 3b shows how the ECGbox builds the packets for the transmission to the laptop. Every 5 ms, the ECGbox builds a packet composed by a unique header (2 bytes), two 12-bit data acquired from the two ECG channels $ECG_{1H,L}$ and $ECG_{2H,L}$ (4 bytes), the bytes coming from SPRbox A channel $SPR_{AH}$ and $SPR_{AL}$ (2 bytes) and the ones coming from SPRbox B channel $SPR_{BH}$ and $SPR_{BL}$ (2 bytes); since the packets coming from SPR boxes arrive every 40 ms, the SPR data are treated as a first-in first-out stack. This means that, in principle, there is a 40 ms delay between ECG channels and SPR channels—the ECG channels are synchronous with the data transmitted to the laptop since they are acquired directly on the ECGbox, while the SPR data belong to the last transmitted packet which is, roughly, 40 ms delayed. Since the bandwidth of SPR signals is in the order of a few hertz, this delay does not affect the accuracy of data alignment.

The WiFi module chosen for the ECGbox is the USR-IOT USR-WIFI232; the device operates as access point (AP), it is configured to be a client UDP which sends the data to the IP address of the server which is the laptop that plots and saves the traces.

### 2.5. Software Description

**Figure 4:**
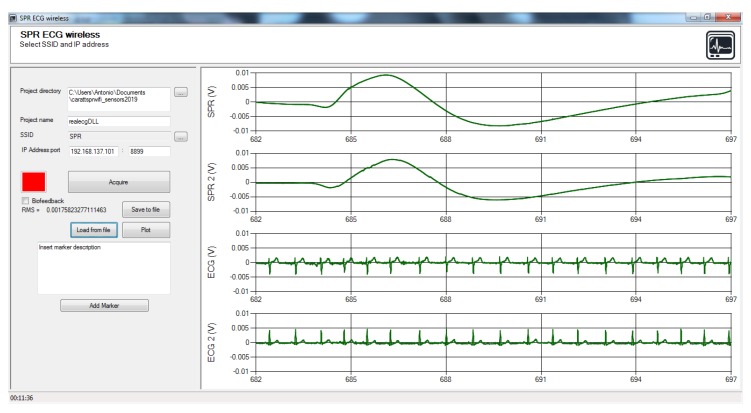
Control panel developed in .NET environment for data acquisition and real time plot; upper traces show SPR channels, bottom traces show ECG channels.

An *Ad hoc* control panel has been designed to acquire, plot and save the data transmitted from ECGbox, Figure 4 shows a screenshot during acquisition.

The panel has been designed in .NET framework since this tool allows a fast and easy design of Graphical User Interfaces (GUI). The GUI communicates with the ECGbox via UDP protocol, extracts the data from the received packets, plots the four signals in real time and allows the insertion of graphical markers with optional comments if the user needs to annotate which kind of stimuli are received by the subject under test. On the left of the GUI there are several controls where the user sets the SSID of the wireless network, the IP address of the server and the folder path where data are saved. The button “Acquire” starts the connection to the ECGbox and the plot of the four traces in real time. The first two graphs on top show the SPR signals, the last two graphs show the two ECG derivations.

## 3. Experimental Results

The sensors system has been characterized with three different approaches and test setups. The first approach characterizes the linearity and the bandwidth of the sensors using, as a reference input, sinusoidal signals and measuring the sensors response at the output. The second approach characterizes the ECG sensor sending at its input a synthesized ECG provided by a digital waveform generator; with this approach we quantify accuracy in detecting R-R intervals at the output with respect to the input. Using a free license commercial software designed to perform HRV analysis [38], we quantify the accuracy of the measured heart rate by means of the jitter introduced by the sensor. The third approach shows the results obtained on real ECG traces acquired simultaneously by the sensor and a commercial one [39], taken as reference; moreover, we quantify the deviation between our sensor and the reference one for the most common HRV parameters used in literature. Finally, at the end of this Section, we show an application example of the proposed sensor measuring the sympathetic activity of drivers in a city traffic simulated scenario.

### 3.1. Metrological Characterization

In this Subsection, we present the experimental characterization of the linearity and bandwidth of the sensor. For the characterization, a GUI has been developed in the Matlab environment to realize an automatic test bench for the acquisition of a large number of samples. An oscilloscope with a built-in waveform generator (RIGOL DS2302A) has been connected to the laptop through LAN and the sensor through WiFi. The test bench GUI controls the waveform generator to generate the appropriate output and a robot code controls the marker insertion and the data saving of the traces transmitted from the sensor (via WiFi) to the control panel described in Section 2.5. At the end of data acquisition, the accuracy of the sensor is evaluated accordingly to the “Guide to the expression of Uncertainty in Measurements” (GUM) [40].

#### 3.1.1. Linearity Characterization and Resolution

For linearity characterization, the waveform generator provided a sinusoidal voltage $V_{G}$ with 50 linearly spaced amplitudes from 0 to 3.3 $V_{PP}$ and with frequency around the centre of the bandwidth for both SPRboxes and ECGbox, i.e. at 1 Hz. The output of the generator is connected to a resistive attenuator α (0.1% tolerance of the resistors) having output impedance 1 MΩ to simulate skin behavior, the value of α has been designed as in [20]. The outputs of the attenuators provide simultaneously the input voltages $V_{IN,SPR}=\alpha_{SPR}V_{G}$ and $V_{IN,ECG}=\alpha_{ECG}V_{G}$ which are connected to the input of SPRboxes and ECGbox. In the case of SPRbox $\alpha_{SPR}=5.568\times 10^{-3}\left(8\times 10^{-6}\right)$, in the case of ECGbox $\alpha_{ECG}=1.497\times 10^{-3}\left(3\times 10^{-6}\right)$. The uncertainty on the input voltages is then calculated using the GUM:

(3) $$u\left(V_{IN,SPR}\right)=\sqrt{{\left[\alpha_{SPR}\cdot u\left(V_{G}\right)\right]}^{2}+{\left[V_{G}\cdot u\left(\alpha_{SPR}\right)\right]}^{2}},$$

(4) $$u\left(V_{IN,ECG}\right)=\sqrt{{\left[\alpha_{ECG}\cdot u\left(V_{G}\right)\right]}^{2}+{\left[V_{G}\cdot u\left(\alpha_{ECG}\right)\right]}^{2}}.$$

In order to evaluate the accuracy of the sensors, the RMS values of the traces plotted on the control panel are evaluated over 10 periods of the input sinusoid; for each amplitude of the sinusoid, 10 RMS values have been acquired (corresponding to the evaluation of 100 periods) in order to evaluate the type A estimation of uncertainty. Thus, at the end of acquisitions, we collect a 50 × 10 matrix for each of the two ECG signals $V_{OUT,ECG}$ and the two SPR signals $V_{OUT,SPR}$.

Then, using the least squares method, we estimated the gains $G_{SPR}$ and $G_{ECG}$ using the input voltage vectors ($V_{IN,SPR}$ and $V_{IN,ECG}$) and the mean of the 10 RMS readouts ($\bar{V_{OUT,SPR}}$ and $\bar{V_{OUT,ECG}}$) calculated on the output voltages vectors.

(5) $$G_{SPR}=\frac{\langle V_{IN,SPR}\bar{V_{OUT,SPR}}\rangle -\langle V_{IN,SPR}\rangle \langle \bar{V_{OUT,SPR}}\rangle }{\langle V_{IN,SPR}{,}^{2}\rangle -{\langle V_{IN,SPR}\rangle }^{2}}$$

(6) $$G_{ECG}=\frac{\langle V_{IN,ECG}\bar{V_{OUT,ECG}}\rangle -\langle V_{IN,ECG}\rangle \langle \bar{V_{OUT,ECG}}\rangle }{\langle V_{IN,ECG}^{2}\rangle -{\langle V_{IN,ECG}\rangle }^{2}}.$$

The uncertainty of the output voltages $u(\bar{V_{OUT,SPR}})$ and $u(\bar{V_{OUT,ECG}})$ is calculated as the combination of two contributions—one is the type A estimation $u_{A}$ obtained as the standard deviation of the sample means over the 10 readings of RMS, and the second is the type B estimation $u_{B}$ extracted from the datasheet of the A/D which considers the Integral Non Linearity (INL) and the quantization uncertainty

(7) $$u\left(\bar{V_{OUT,SPR}}\right)=\sqrt{{\left[u_{A}\left(\bar{V_{OUT,SPR}}\right)\right]}^{2}+{\left[u_{B}\left(\bar{V_{OUT,SPR}}\right)\right]}^{2}},$$

(8) $$u\left(\bar{V_{OUT,ECG}}\right)=\sqrt{{\left[u_{A}\left(\bar{V_{OUT,ECG}}\right)\right]}^{2}+{\left[u_{B}\left(\bar{V_{OUT,ECG}}\right)\right]}^{2}}.$$

We recall that $V_{IN,SPR}$ and $V_{IN,ECG}$ are vectors composed by 50 test amplitudes and that $\bar{V_{OUT,SPR}}$ and $\bar{V_{OUT,ECG}}$ are vectors obtained as the mean of 10 readings for each output amplitude. Finally, combining (3)–(8) and naming with subscript *i* the input and output voltages at the *i*th step, we calculate the uncertainty on the gain as

(9) $$u\left(G_{SPR}\right)=\sqrt{\sum_{i=1}^{50}{\left(\frac{\partial G_{SPR}}{\partial V_{IN,SPR,i}}u\left(V_{IN,SPR,i}\right)\right)}^{2}+\sum_{i=1}^{50}{\left(\frac{\partial G_{SPR}}{\partial \bar{V_{OUT,SPR,i}}}u\left(\bar{V_{OUT,SPR,i}}\right)\right)}^{2}},$$

(10) $$u\left(G_{ECG}\right)=\sqrt{\sum_{i=1}^{50}{\left(\frac{\partial G_{ECG}}{\partial V_{IN,ECG,i}}u\left(V_{IN,ECG,i}\right)\right)}^{2}+\sum_{i=1}^{50}{\left(\frac{\partial G_{ECG}}{\partial \bar{V_{OUT,ECG,i}}}u\left(\bar{V_{OUT,ECG,i}}\right)\right)}^{2}}.$$

**Figure 5:**
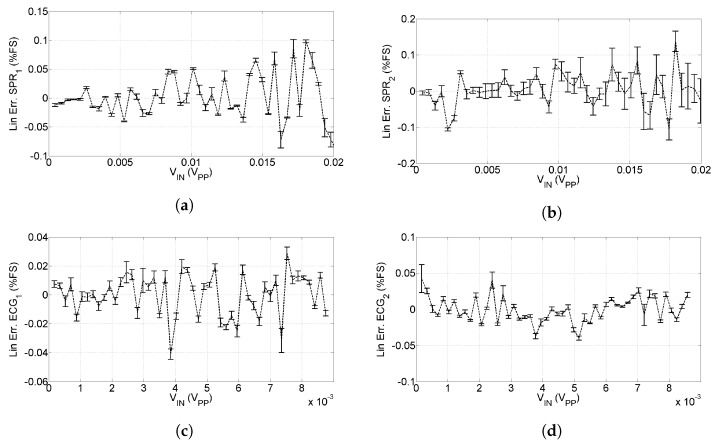
Linearity of the developed sensors, error bars represent the uncertainty on linearity. (**a**) Linearity for *SPR*_1_ signal, (**b**) Linearity for *SPR*_2_ signal, (**c**) Linearity for *ECG*_1_ signal, and (**d**) Linearity for *ECG*_2_ signal.

The gains of the four channels thus result in $G_{SPR1}=141.0\pm 0.6$, $G_{SPR2}=141.2\pm 0.6$, $G_{ECG1}=378.3\pm 1.6$ and $G_{ECG2}=376.9\pm 1.6$. After having characterized the gain of the four channels, we are interested in investigating the deviation from linearity. Figure 5 shows the relative deviation from linearity with respect to the full scale of each input.

As can be seen, the maximum non-linearity for the SPR channels is 0.15% FS, corresponding to 30μV and the maximum non-linearity for the ECG channels is 0.05% FS, corresponding to 5μV; error bars in Figure 5 represent the uncertainty contributions evaluated in (3), (4), (7) and (8).

The resolution of the sensor is 12 bit, which corresponds to 4.9μV for SPR channels and 2.4μV for ECG channels.

#### 3.1.2. Bandwidth Characterization

For bandwidth characterization, the waveform generator provided a sinusoidal voltage with 61 logarithmically spaced steps in the range [0.1,100] Hz and the amplitude of the generator was set to 3.3 $V_{PP}$; the output of the generator has been connected to the same attenuator described in previous Section. With the same GUI with robot code described in the previous Section, we acquired the outputs for each frequency step over a variable time window W=min(10periods,3s); in this way, we compute the gains $G_{SPR}\left(j\omega \right)$ and $G_{ECG}\left(j\omega \right)$ observing the signals for a duration of 10 periods (coherent sampling) when the frequency is low, and for a duration of three seconds when the frequency is high.

**Figure 6:**
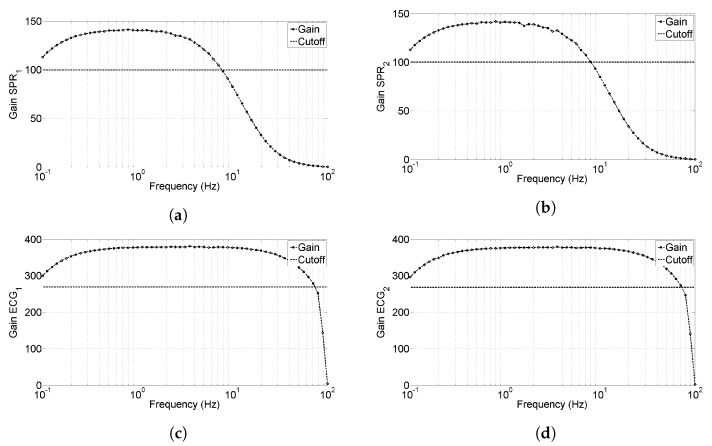
Bandwidth characterization for the developed sensors, horizontal line represents the cut-off limit. (**a**) Bandwidth for *SPR*_1_ signal, (**b**) bandwidth for *SPR*_2_ signal, (**c**) bandwidth for *ECG*_1_ signal, and (**d**) bandwidth for *ECG*_2_ signal.

Figure 6 shows the magnitude of the gain for each channel; the upper cut-off frequency results 8 Hz and 75 Hz for SPR channels and ECG channels, respectively. Regarding the lower cut-off, it results lower than 0.1 Hz which is the limit of the waveform generator (from design it is expected 0.08 Hz).

#### 3.1.3. Delays in Transmission and Data Loss

As introduced in Section 2.4, the SPR data are available at ECGbox after that the packet, composed by 8 SPR data, is sent from the SPRbox. This means that SPR data are delayed with respect to the ECG ones and, if no data loss happened, the delay should be in the order of 40 ms. In order to characterize the real delays of transmission, we used the data acquired in Section 3.1.1 (duration of acquisition 1120 s corresponding to 224,000 samples per channel) to characterize the average delay from one trace to another. Since all the inputs are in phase, we compute the Fast Fourier Transform (FFT) of each signal and we evaluate its phase on the peak (that is at 1 Hz); then, we calculate the difference of phases from one channel to another and convert it into a time delay. Naming $\Delta t_{j,k}$ the delay between signal *j* and *k*, we obtained $\Delta t_{SPR1,SPR2}=6\;\mathrm{ms}$, $\Delta t_{SPR1,ECG1}=43\;\mathrm{ms}$, $\Delta t_{SPR1,ECG2}=43\;\mathrm{ms}$, $\Delta t_{SPR2,ECG1}=49\;\mathrm{ms}$, $\Delta t_{SPR2,ECG2}=49\;\mathrm{ms}$, $\Delta t_{ECG1,ECG2}=72\;\mu s$. From these results, we can see that the delays are comparable with the values expected by design—$ECG_{1}$ and $ECG_{2}$ are substantially synchronous, while SPR channels are 43 to 49 ms delayed. As stated before, this delay is negligible since the SPR signal bandwidth is in the order of few hertz. Regarding data loss, we have to notice that no data have been lost in communication from ECGbox to laptop; from SPRbox to ECGbox instead, we counted 98 samples that were belonging to the previous packet, so we can say that the data loss from SPRbox to ECGbox is of 98 samples over a total amount of 224,650 samples, corresponding to the 0.04% (i.e., on average, one sample lost every 12 s of acquisition).

### 3.2. Jitter Estimation Using Synthesized ECG

**Figure 7:**
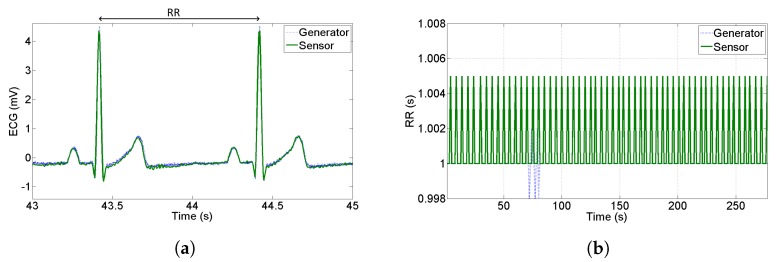
(**a**) Comparison between the synthesized peaks acquired from the oscilloscope and the proposed sensor, and (**b**) tachogram extraction to evaluate the jitter introduced by the sensor.

In the perspective of using ECG data to perform HRV analysis, it is very important to quantify how accurate the recognition of R-peaks is; one of the main contributions to accuracy is the jitter introduced by the sample rate and bandwidth of the sensor. In this Subsection, we build the RR-tachogram starting from a synthesized ECG provided by an arbitrary waveform generator. We acquire simultaneously the output of the sensor and the output of the generator which was set to provide an ECG with R-R peaks at constant distance 1 s (1 Hz frequency corresponding to 60 bpm). Figure 7a shows a qualitative comparison between the PQRST complex at the generator and at the output of the proposed sensor—no attenuation, delay or distortions are noticed. On the other hand, Figure 7b shows the RR intervals estimation starting from the generator and starting from the proposed sensor. The RR tachogram has been built acquiring the signal for a duration of 5 minutes and using the commercial software Kubios HRV which implements the Pan Tompkins algorithm [41] for peak recognition. As can be seen in Figure 7b, the maximum distance of computed RR is 5 ms with respect to the nominal value—this means that the R-peaks have been detected with deterministic uncertainty equal to the sample period. The standard deviation of the tachogram obtained by the sensor results 2 ms, so we can conclude that the jitter introduced by the sensor is very close to the theoretical one that is $5/\sqrt{3}=2.8$ ms, assuming a uniform distribution of deterministic uncertainty.

### 3.3. Real ECG Acquisitions and Comparison with Benchmark Device

**Figure 8:**
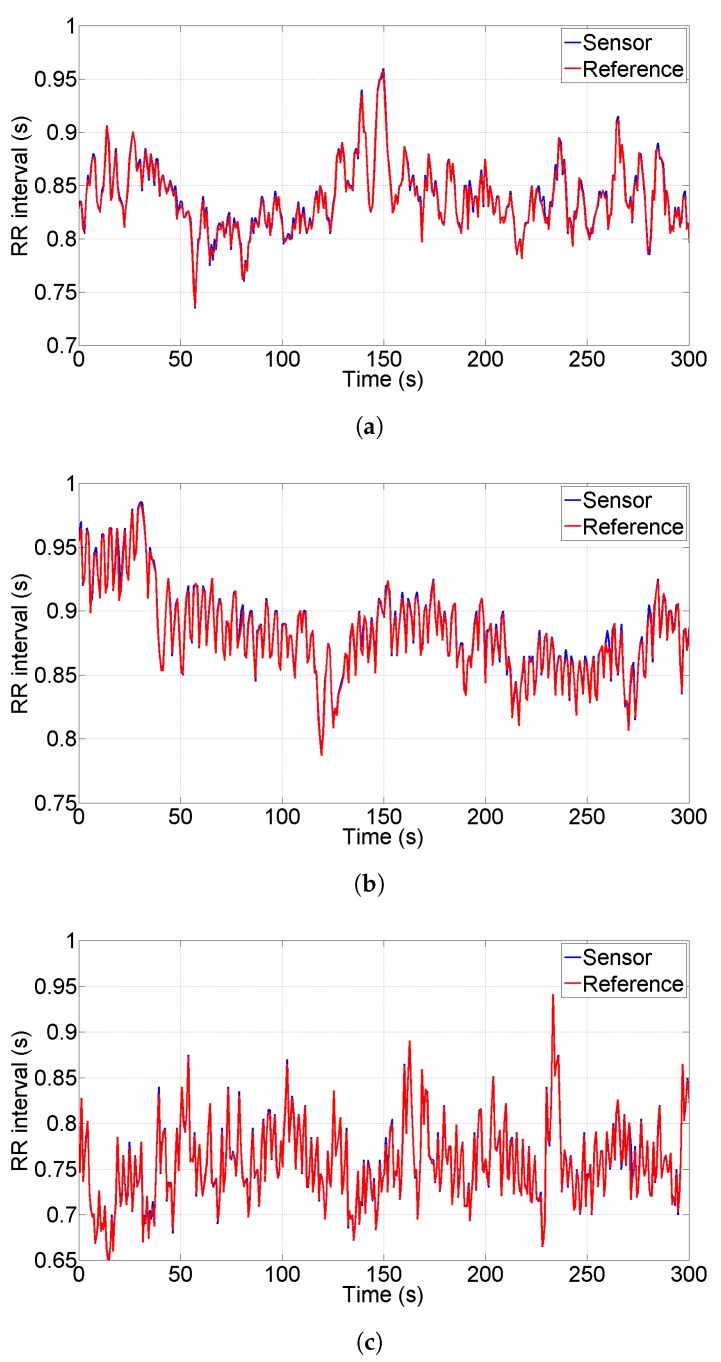
Comparison of Tachograms extracted acquiring data with the proposed sensor and with a reference one on three different individuals (**a**–**c**): blue lines represent the proposed sensor, red lines represent the reference one.

As a third approach for sensor characterization, in this Subsection we compare the tachogram obtained from the proposed sensor with the one obtained from a commercial sensor used as reference. We acquired the data simultaneously placing the reference sensor electrodes close to the derivation $V_{4}-V_{1}$ shown in Figure 1. The reference sensor is a Shimmer 3 EXG unit and it has been configured to acquire data at sample rate 512 Sa/s. We chose the Shimmer EXG as reference because of its compactness, of its high performances (sampling rate up to 8 kSa/s) and of its reasonable cost (one Shimmer sensor costs ten times the sensor presented in this paper). We collected a total number of 18 tests performed on three different individuals (six tests per person) acquiring data for a duration of five minutes per test, this duration is typical for HRV analysis. Figure 8a–c show the tachograms extracted for each person under test; blue lines represent the proposed sensor, red lines represent the reference instrument. As a qualitative analysis, we observe that all the R-peaks have been detected without artifacts and the two lines are almost indistinguishable for all the three persons; in particular, the standard deviation of the difference between the two plotted lines is 4 ms, 5.4 ms and 3.6 ms, respectively.

In order to perform a quantitative analysis, we processed the data saved from both sensors using Kubios and we evaluated the most common parameters used in HRV analyses. In particular, we focused our attention to mean HR, SDNN, RMSSD, LF and HF. Table 1 shows the results obtained from both sensors.

In order to evaluate the deviation between the proposed sensor and the reference, we present in Table 2 the relative difference between the two sensors in the estimation of HRV parameters. From the data, emerges that the maximum deviation is in the order of 6%.

### 3.4. Acquisition of SPR and ECG Signals on a Driving Simulator

**Figure 9:**
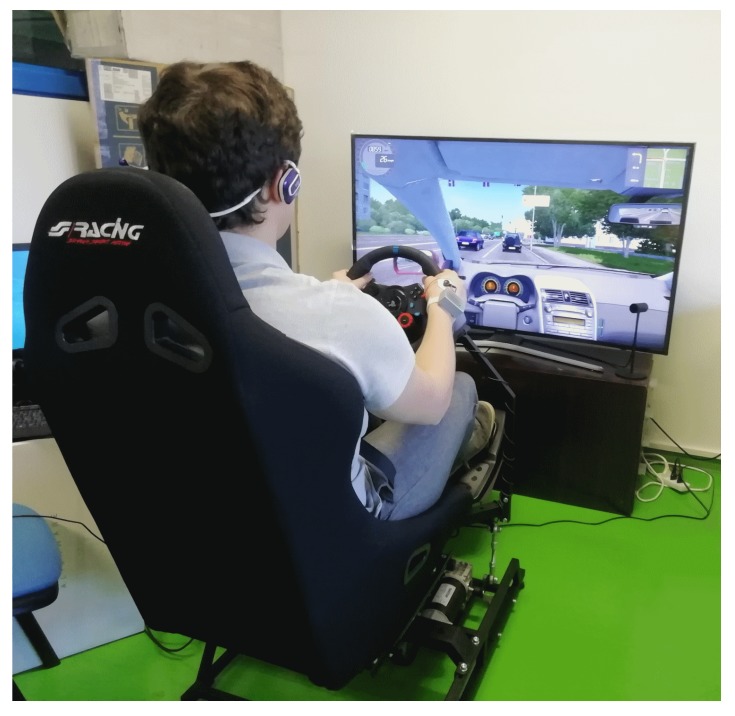
The driving simulator available in our laboratory at the University of Udine.

As an application example of the proposed sensor, we performed several tests on the driving simulator available in our laboratory at the Polytechnic Department of Engineering and Architecture of the University of Udine. The simulator is composed by the moving seat P2 by DOFreality [42], a curved screen and a Logitech G29 Steering Wheel [43], Figure 9 shows a picture of the simulator.

As simulator software, we use City Car Driving; it is a software simulating traffic situations in a city with the option to insert, as stressors, a set of undesired events like: unexpected pedestrian crossing, lane invasion by other vehicles in the opposite direction, sudden change of lane of the vehicle in front of the driver, and sudden brake of the front vehicle. As a limitation, this software does not allow the full control on other vehicles or pedestrian behavior (platoons of cars are not the same, pedestrians do not cross always in the same locations...), but the type and the number of stressors among different simulations is consistent, accordingly to the settings of the simulation.

**Figure 10:**
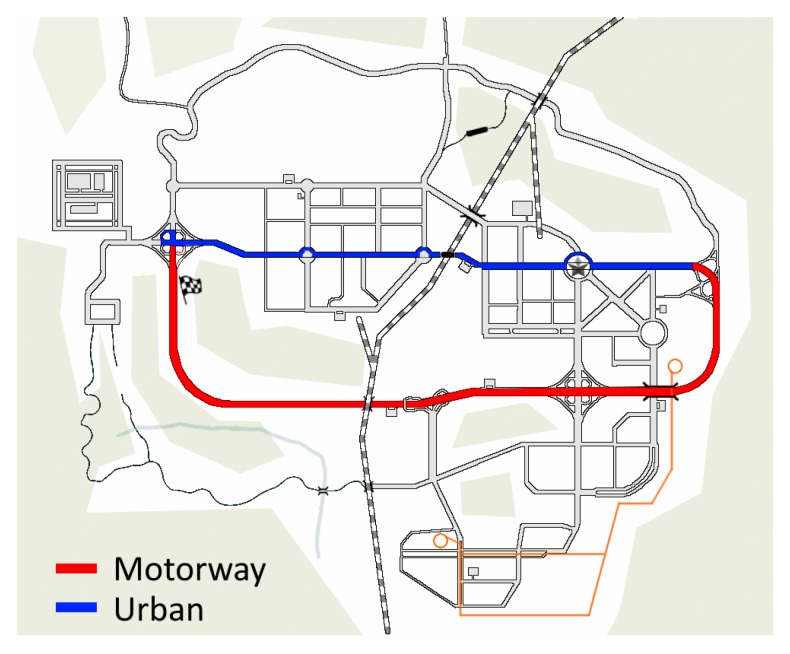
City map of the driving experiments; red line represents the motorway, blue line represents urban streets and checkered flag represents the start and the stop of experiments.

We instructed ten volunteers (among the students of University of Udine) to drive along the route highlighted in Figure 10; the red line represents a portion of a motorway, while the blue line represents urban streets. The subjects had to repeat the test in two conditions—a first condition is without traffic (no cars nor pedestrians) and the second condition is with few cars and pedestrians. In this second scenario, despite little traffic, we set up the behavior of other cars and pedestrians as “very aggressive”, this means that other cars often change their direction (invading the subject’s trajectory) and pedestrians very often cross the road at forbidden points. One half of the subjects drove before without traffic and then with traffic and the other half vice-versa. The selected path on the map in Figure 10 allowed the recording of ten minutes traces for each experiment, roughly one half in motorway and one half in urban streets.

**Figure 11:**
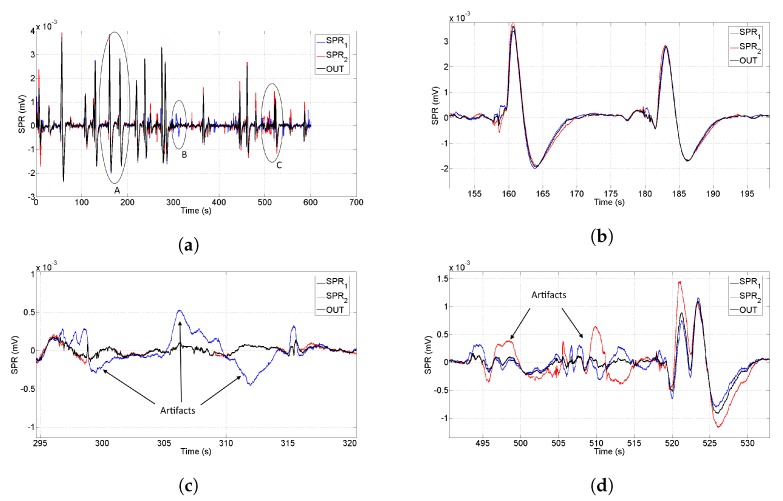
(**a**) Acquired SPR signals (blue line *SPR*_1_ on right hand, red line *SPR*_2_ on left hand) and output of the algorithm for motion artifact removal (black line) during a driving session; circles A, B and C represent some significant portions zoomed in the next sub-figures. (**b**) Zoom of the signals in the circle A; when hands are still, the three traces are identical and SPR pulses look with smooth shape. (**c**) Zoom of the signals in the circle B; it is evident a motion artifact in right hand, but the output is not affected remaining close to zero. (**d**) Zoom of the signals in the circle C; motion artifacts are evident in both hands in *t* ∈ [495, 515] s, but the output is not affected; at *t* = 520 s instead, an SPR pulse is properly recognized.

Regarding the processing of SPR data, we applied the motion artifact algorithm described in Reference [44]; for ease of reference, we show in Figure 11a the acquired traces (blue line $SPR_{1}$ for right hand and red line $SPR_{2}$ for left hand) and the output of the motion artifact removal algorithm (black line). Figure 11b shows the zoom of circle A in Figure 11a and we show the typical SPR pulses that we have when no motion artifacts are intervening; all the traces are almost coincident and each SPR peak duration is, roughly 10 s. The circles marked with letters B, and C in Figure 11a evidence the motion artifacts that arose when the hands were moving the steering wheel; in that case, the two input signals differed and some high frequency components appeared. Figure 11c,d show the zoom of circles B and C; as it can be seen, at the output the artifacts are strongly reduced.

Since in this experiment the subjects were driving on a motorway or in a city (and not in a race track), the artifacts appeared very rarely (the hands motion was limited) and the algorithm removed all the possible artifacts that were appearing in the signals.

After the artifact removal, we evaluated the Root Mean Square (RMS) value over a moving window having duration 5 s. Regarding ECG traces, we extracted several features like HR, SDNN, LF and LF/HF.

**Figure 12:**
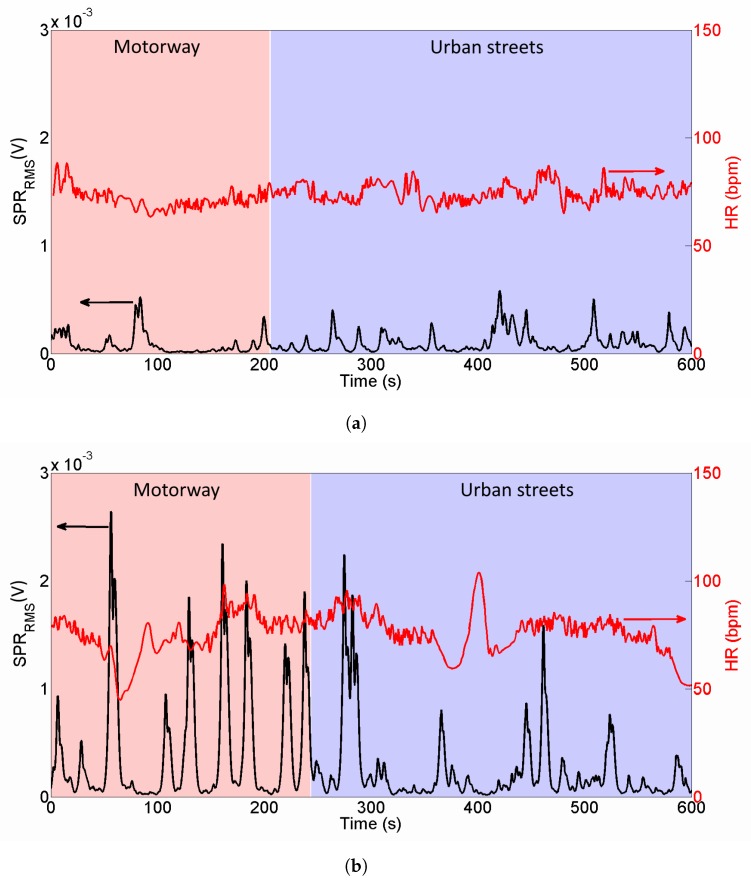
Acquired traces from a subject driving without traffic (**a**), and with aggressive traffic (**b**). Black line (left axis) represents *SPR_RMS_* signal, red line (right axis) represents *HR* signal. It is evident that SPR peaks are by far higher during traffic test and HR shows a higher variability.

Figure 12a shows the extraction of $SPR_{RMS}$ signal (black line, left axis) from the trace in Figure 11a and HR signal (red line, right axis) during the driving without traffic. Pink background represents motorway, light blue background represents urban streets. With the same axes scales, Figure 12b represents the traces of the same subject when driving with aggressive traffic.

From Figure 12 it is evident that SPR activity is considerably higher when driving in aggressive traffic—the height of the peaks in Figure 12b are higher than the ones in Figure 12a and every peak of black line corresponds to a sudden lane change of other vehicles or a pedestrian crossing. Similarly, it is possible to notice that HR has increased variability during a traffic test.

In order to provide a quantitative estimation of perceived stress, we report in Table 3 the mean values of $SPR_{RMS}$, HR, SDNN, LF and LF/HF for each driver and for each experiment. Regarding SPR activity, it is possible to observe that all the subjects show a higher $SPR_{RMS}$ during the traffic test; in particular, they present an average 39% increase of SPR activity in a traffic test with respect to a no-traffic drive test. Regarding HRV, we notice that HR increases in 8 subjects out of 10, with 2% average increase, SDNN increases in 6 subjects out of 10, with 19% average increase; LF increases in 6 subjects out of 10, with 14% average increase and LF/HF increases in 8 subjects out of 10, with 42% average increase.

Performing a paired *t*-test between the two sets of data (no traffic and aggressive traffic), we observe that the $SPR_{RMS}$ is significantly higher with traffic (p=0.04), HR is significantly higher with traffic (p=0.03) and LF/HF is almost significantly higher with traffic (p=0.06). On the other hand, we observe that SDNN and LF are higher with traffic but with low significance (p=0.22 for both features).

Performing a non-parametric test (Wilcoxon signed rank test) between the two sets of data, we observe results similar to *t*-test. In particular, the test on $SPR_{RMS}$ provides p=0.002, thus the traffic condition provides higher SPR with high significance; on HR the test provides p=0.03 so the traffic condition provides higher HR with significant difference; LF/HF is almost significantly higher with traffic (p=0.08) while SDNN and LF have lower significance (p=0.13 and p=0.27, respectively).

The results from both *t*-test and Wilcoxon test remark two important aspects—(1) the necessity of evaluating more than a single parameter in HRV—for example, evaluating the quantity SDNN·LF/HF, the Wilcoxon test provides p=0.02; (2) the importance of acquiring SPR and HRV simultaneously (any combination of SPR with HRV parameters provides p<0.05).

In this scenario, it emerges that the simultaneous acquisition of SPR+ECG with a unique timebase and with a fully wireless sensor system can be a valuable help to the research in this field.

## 4. Discussion

From the characterization described in previous Section, we can observe several interesting points regarding the accuracy of the developed sensor system.

Regarding the characterization of gain and bandwidth shown in Section 3.1, we have to point out that the gains of the two ECG channels and of the two SPR channels present a very small mismatch and all the channels present a non-linearity in the order of 0.1%. This confirms the high level of accuracy of the developed system; moreover, the delay between local signals (ECG) and transmitted ones (SPR) has been validated in the order of 40 ms, as estimated during the design phase. Finally, the bandwidths of the analog front ends respect the initial specifications.

The jitter estimation with synthesized ECG demonstrated two important aspects. First, the chosen analog bandwidth of 75 Hz does not alter the shape of R-peaks (shown in Figure 7a) and thus it is suitable for tachogram extraction. Second, the jitter introduced by the sensor results lower than sample rate, thus its effect is negligible for HRV estimation.

The comparison of ECG acquired with a reference sensor points out some problematic aspects of HRV estimation. First, looking at Table 2, we observe that the extraction of mean heart rate is extremely accurate (in the order of 0.2%); the SDNN (in time domain) and the LF (in frequency domain) provide quite accurate results, in the order of 2%, whilst the least accurate results are provided by the RMSSD and HF (6%). Observing the data in Table 1 we can see that the magnitude of HF and RMSSD is quite small in all the acquired experiments (sympathetic activity prevailing on the vagal) and thus, a small error in absolute terms can reach significant values in relative terms. This observation keeps still open the issue that, even if HR is evaluated with high accuracy, the HRV has a strong dependency on very small variations; for this reason the simultaneous acquisition of ECG and EDA can be a useful tool to improve the knowledge of HRV.

## 5. Conclusions

We designed a wearable sensors system for the simultaneous measurement of two channels SPR acquired on the hands and two channels ECG acquired on the chest. The SPR data are sent via WiFi to the ECG box so the system is completely wireless in order to achieve increased wearability.

The characterization showed high-level performances in terms of linearity and jitter even if the SPR channels are not wired, despite of a small delay accounted during the design process.

The developed sensor is suitable for stress detection in drivers at simulators, but its application can be extended to other fields like safety on workplace, game addiction, development of adaptive video games, and so on.

As a future work, we are studying an improvement of the motion artifact algorithm presented in Reference [44]; the main limit of this methodology, in fact, is the accurate removal of motion artifacts that arise when the subject, with sudden and rough movements, solicits the electrode posed on the palm of the hand.

We are also working to improve the SVM described in Reference [26], in particular we want to discriminate the minimum set of features to accurately train the SVM, and we are going to integrate the system with computer vision sensors measuring the pupil diameter, eye blinking rate and the gaze direction.

## Figures and Tables

**Table 1:** Comparison between developed sensor and reference for principal heart rate variability (HRV) features.

Test #	Sensor					Reference				
	HR	SDNN	RMSSD	LF	HF	HR	SDNN	RMSSD	LF	HF
	(bpm)	(ms)	(ms)	(ms${}^{2}$)	(ms${}^{2}$)	(bpm)	(ms)	(ms)	(ms${}^{2}$)	(ms${}^{2}$)
1	75.903	40.916	20.242	488.31	91.864	76.013	40.742	19.468	496.39	87.125
2	74.693	43.338	18.355	628.14	72.580	74.811	43.176	17.673	627.78	70.115
3	74.657	41.674	17.148	363.47	86.098	74.774	41.406	16.386	366.41	81.716
4	73.482	47.612	18.186	222.03	106.33	73.599	47.453	17.537	221.73	101.75
5	71.981	32.295	17.707	336.96	74.507	72.102	32.201	17.203	333.97	70.242
6	71.73	31.691	18.392	476.8	67.139	71.852	31.623	18.161	476.05	67.896
7	68.054	34.675	22.568	134.18	177.7	68.152	34.505	22.675	136.87	184.82
8	68.54	26.808	21.664	93.33	171.51	68.652	26.716	21.938	92.85	172.08
9	68.976	25.858	22.917	91.28	191.59	68.791	25.231	21.547	91.05	188.16
10	68.751	28.581	24.007	83.69	179.47	68.867	27.93	22.55	87.25	181.5
11	68.331	28.855	24.293	128.52	199.05	68.454	28.254	22.725	124.42	195.23
12	68.073	26.624	23.802	189.73	240.78	68.198	26.179	22.473	181.39	227.37
13	79.857	43.445	40.166	626.98	508.68	79.97	43.421	40.547	619.72	504.26
14	79.582	43.674	39.696	761.04	558.41	79.7	43.578	39.947	758.69	556.97
15	79.564	48.758	41.529	749.02	532.59	79.689	48.668	41.682	746.43	523.12
16	78.98	49.145	41.644	1000.9	614.16	79.105	48.97	41.45	984.13	597.78
17	79.196	48.47	40.791	958.95	894.56	79.323	48.154	40.2	950.35	861.06
18	79.012	49.28	42.327	632.74	826.41	79.137	48.978	41.624	636.95	778.08

**Table 2:** Relative deviation between developed sensor and reference for principal HRV features.

Test #	Δ%				
	HR	SDNN	RMSSD	LF	HF
1	0.14	−0.43	−3.9	1.6	−5.4
2	0.16	−0.38	−3.8	−0.06	−3.5
3	0.16	−0.65	−4.6	0.80	−5.3
4	0.16	−0.34	−3.7	−0.14	−4.5
5	0.17	−0.29	−2.9	−0.90	−6.1
6	0.17	−0.22	−1.2	−0.16	1.1
7	0.14	−0.49	0.47	1.9	3.8
8	0.16	−0.34	1.2	−0.52	0.33
9	−0.27	−2.5	−6.3	−0.25	−1.8
10	0.17	−2.3	−6.4	4.1	1.1
11	0.18	−2.1	−6.9	−3.3	−1.9
12	0.18	−1.7	−5.9	−4.6	−5.9
13	0.14	−0.06	0.94	−1.2	−0.88
14	0.15	−0.22	0.63	−0.31	−0.26
15	0.16	−0.18	0.37	−0.35	−1.8
16	0.16	−0.36	−0.47	−1.7	−2.7
17	0.16	−0.66	−1.4	−0.90	−3.8
18	0.16	−0.62	−1.6	0.66	−6.2
Mean	0.13	−0.77	−2.5	−0.29	−2.4

**Table 3:** Comparison between SPR and HRV features extracted in no-traffic or traffic scenarios.

Subject #	No-Traffic					Traffic				
	${\mathrm{SPR}}_{\mathrm{RMS}}$	HR	SDNN	LF	LF/HF	${\mathrm{SPR}}_{\mathrm{RMS}}$	HR	SDNN	LF	LF/HF
	(mV)	(bpm)	(ms)	(ms${}^{2}$)		(mV)	(bpm)	(ms)	(ms${}^{2}$)	
1	0.271	96.9	25.17	333	6.11	0.379	96.1	32.51	502	12.2
2	0.651	97.5	41.62	455	5.01	0.773	97.6	39.62	405	5.61
3	0.307	74.3	44.71	937	4.69	0.434	75.8	52.73	918	3.14
4	0.571	75.9	66.21	831	4.71	0.653	75.4	63.84	1499	8.64
5	1.5	71.4	52.31	1129	3.74	1.6	72.1	57.82	1352	4.17
6	0.092	73.9	50.81	442	2.25	0.299	76.3	114.3	586	3.04
7	0.124	67.8	45.51	770	3.75	0.921	71.9	52.61	861	2.55
8	0.326	80.9	34.71	631	3.57	0.354	82.8	31.62	391	6.55
9	0.191	79.9	42.31	798	2.24	0.223	84.7	36.42	830	4.62
10	0.186	78.4	37.42	813	2.07	0.241	79.6	41.85	783	3.48
Mean	0.422	79.7	44.07	714	3.81	0.587	81.2	52.33	813	5.41
